# Nurse burnout before and during the COVID-19 pandemic: a systematic comparative review

**DOI:** 10.3389/fpubh.2023.1225431

**Published:** 2023-09-05

**Authors:** Amelia Rizzo, Murat Yıldırım, Gülçin Güler Öztekin, Alessandro De Carlo, Gabriella Nucera, Łukasz Szarpak, Salvatore Zaffina, Francesco Chirico

**Affiliations:** ^1^Department of Clinical and Experimental Medicine, University of Messina, Messina, Italy; ^2^Department of Psychology, Faculty of Science and Letters, Agri Ibrahim Cecen University, Agri, Türkiye; ^3^Department of Emergency, Fatebenefratelli Hospital, ASST Fatebenefratelli and Sacco, Milan, Italy; ^4^Institute of Outcomes Research, Maria Sklodowska-Curie Medical Academy, Warsaw, Poland; ^5^Research Unit, Maria Sklodowska-Curie Bialystok Oncology Center, Bialystok, Poland; ^6^Henry JN Taub Department of Emergency Medicine, Baylor College of Medicine, Houston, TX, United States; ^7^Occupational Medicine Unit, Bambino Gesù Children's Hospital, Scientific Institutes for Research and Care (IRCCS), Rome, Italy; ^8^Post-Graduate School of Occupational Health, Università Cattolica del Sacro Cuore, Rome, Italy; ^9^Health Service Department, Italian State Police, Ministry of the Interior, Centro Sanitario Polifunzionale, Milan, Italy

**Keywords:** COVID-19 pandemic, nurse burnout, systematic review, comparative analysis, healthcare professionals

## Abstract

**Introduction:**

This review aimed to compare available evidence examining burnout using the Maslach Burnout Inventory (MBI) in nurses before and during the COVID-19 pandemic. The specific objective was to compare nurse burnout scores in terms of emotional exhaustion, depersonalization, and personal accomplishment.

**Methods:**

A comprehensive search was conducted for studies on nurses' burnout using the MBI published between 1994 and 2022. In total, 19 studies conducted prior to the pandemic and 16 studies conducted during the pandemic were included and compared using the criteria from the Joanna Briggs Institute Critical Appraisal Tool.

**Results:**

Surprisingly, the results indicated that nurses' burnout scores did not differ significantly before (*N* = 59,111) and during (*N* = 18,629) the pandemic. The difference observed was qualitative rather than quantitative.

**Discussion:**

The outbreak of the COVID-19 pandemic exacerbated an already critical situation, and while COVID-19 may serve as an additional triggering factor for staff mental illness, it cannot solely explain the observed burnout levels. These findings underscore the need for long-term clinical and preventive psychological interventions, suggesting that psychological resources should not be limited to emergencies but extended to address the ongoing challenges faced by nurses.

**Systematic review registration:**

https://www.crd.york.ac.uk/PROSPERO/display_record.php?RecordID=399628, identifier: CRD42023399628.

## 1. Introduction

A major problem worldwide is the shortage of medical personnel. As early as 2006, the World Health Organization reported that the shortage of nurses and health workers has a significant negative impact on improving the health and wellbeing of the world's population (1). The nursing profession in many countries faces increased rates of burnout caused by unrealistic expectations of work, poor working conditions, work demands that exceed personal resources, poor relationships between professionals, and ultimately an increase in occupational health risks (2).

Burnout is characterized by a relatively rapid decline in emotional, physical, and psychological energy as a result of increased work stress. It often leads to a sense of low self-efficacy and results from work overload, a lack of control, resources, and equity. It can also be caused by a lack of community and value conflicts (3). The psychological phenomenon of burnout usually consists of three main factors as follows: emotional exhaustion (EE), i.e., emotional and physiological exhaustion due to work stress, leading to a decrease in energy, fatigue, despair, depression, and helplessness; depersonalization (DP), which refers to negative and insensitive behavior toward others and detachment from the needs of others and guidelines; and finally, a sense of low personal accomplishment (PA), i.e., an evaluation of oneself as inadequate and failing (4). These features of burnout lead to increased turnover rates and have a negative impact on the quality of healthcare.

This has been explained by the concepts of “compassion fatigue” and “caring burden” (5, 6) as the health profession requires a high level of relational and empathic engagement (7). It has been found that burnout in nurses is often associated with a deterioration in physical wellbeing, psychosomatic symptoms, such as insomnia, and psychological symptoms, such as depression. The discomfort is first felt in the professional sphere but then easily transfers to the personal level, and alcohol and psychoactive substance abuse and the risk of suicide are high among burnout sufferers (8).

The COVID-19 outbreak has created unique stressors and challenges, especially for frontline nurses. These stressors and challenges include moral and ethical issues (9). In Italy, Damico et al. (10) found a prevalence of burnout-related symptoms observed in at least 68% of nurses: 77.4% were at risk for EE, 68.7% for DP, and 77.9% for decreased PA. In addition, a statistically higher risk was observed among nurses in COVID-19 wards for EE (54.4 vs. 30.6%, *p* < 0.01), DP (39.7 vs. 23.6%, *p* = 0.019), and decreased PA (44.8 vs. 29.2%, *p* = 0.027), suggesting that the risk of burnout was lower in nurses who did not care for patients with COVID-19 during the emergency.

Despite this evidence, critical levels of burnout in pre-pandemic nurses were found in the literature among different types of units (11, 12). Previous studies and reviews found no differences in burnout, EE, DP, or PA between chronic and acute units. Some authors argue that the level of nurses' dissatisfaction may be related to increased workload combined with a reduction in relationship time (13). Considering EE as an isolated factor, it is significantly higher among nurses in the emergency department; DP, on the other hand, was not found in this area but showed very high scores in chronic units (14).

The international literature has focused heavily on burnout in healthcare workers in the last 2 years, leading to possible biases, such as the association of burnout with the COVID-19 pandemic. To our knowledge, an estimate of the difference between burnout levels in nurses before and during the COVID-19 pandemic is lacking. The aim of this review is, therefore, to compare studies that looked at burnout levels in its subcomponents EE, DP, and PA among nurses before and during the COVID-19 outbreak.

## 2. Materials and methods

### 2.1. Instruments

The Maslach Burnout Inventory (MBI) is the most well-known and extensively used instrument for evaluating burnout. The theoretical foundations of the MBI are based on the tri-dimensional model of burnout by Maslach (15), comprising “exhaustion,” “cynicism,” and “ineffectiveness.” Maslach's model includes precise definitions for each dimension that align well with the corresponding measurement tool. There are currently five versions of the MBI as follows: (1) Human Services Survey (MBI-HSS), (2) Human Services Survey for Medical Personnel [MBI-HSS (MP)], (3) Educators Survey (MBI-ES), (4) General Survey (MBI-GS), and (5) General Survey for Students [MBI-GS (S)].

In a study conducted according to MOOSE (meta-analysis of observational studies in epidemiology) and the PRISMA guidelines recommended by the Cochrane Collaboration (16), the degree of burnout is taken into account (Table 1).

**Table 1:** MBI scoring guide.

**Burnout construct**	**Cutoff score**		
EE	0–18	19–26	>27
	Low	Moderate	High
DP	0–5	6–9	>10
	Low	Moderate	High
PA	0–33	34–39	>40
	High	Moderate	Low

EE, emotional exhaustion; DP, depersonalization; PA, personal accomplishment (17).

### 2.2. Procedure

#### 2.2.1. Protocol registration

The systematic review and meta-analysis were conducted according to the protocol of the International Platform for Registered Systematic Reviews and Meta-Analyses. This study is based on the development of published data; therefore, ethical approval is not required. The study protocol was registered on PROSPERO, the International Prospective Register of Systematic Reviews, National Institute of Health Research, University of York, with the registration number “CRD42023399628.”

#### 2.2.2. Literature search strategy

The analysis was conducted according to the steps of the Preferred Reporting Items for Systematic Reviews and Meta-Analyses (PRISMA) procedure (see flowchart). To identify relevant studies, we searched the Web of Science, PubMed, Medline, and the CINAHL Plus (Cumulative Index of Nursing and Allied Health Literature) databases. Detailed search terms were a combination of “nurse,” “burnout,” “burnout syndrome,” “MBI,” “Maslach Burnout Inventory,” and “COVID-19.” Studies that examined burnout in nurses using the MBI were collected from 1994 to 2022. All those studies that had used one of the other versions of the scale were excluded; studies that did not report mean and standard deviations, but other indices related to burnout, such as correlations, positive case rate, and prevalence were also excluded. To ensure that no relevant articles were missed, the researchers of this study independently searched the reference lists of the included studies.

#### 2.2.3. Study selection process and eligibility criteria

**Figure 1:**
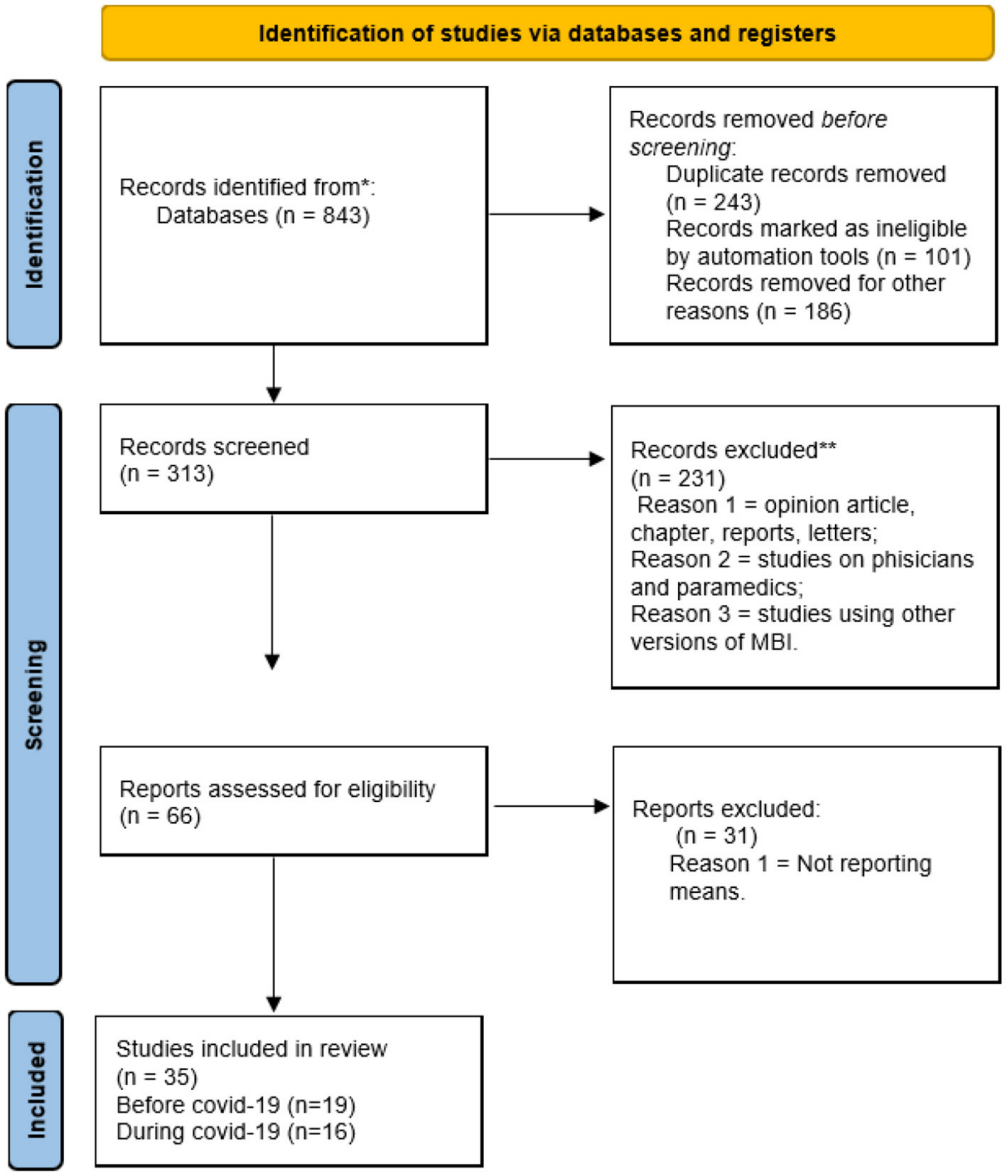
PRISMA 2020 flow diagram for systematic review.

PRISMA was used to select the relevant studies. The words “Maslach Burnout Inventory” and nurse were searched. A total of 843 results were produced, of which 105 were produced in 2020, 140 in 2021, and 127 in 2022, while in previous years, there were < 100 studies per year. A total of 530 studies were excluded before screening because of duplication (*n* = 243) and were marked as ineligible by automation tools (*n* = 101) and other reasons (*n* = 186). The remaining 313 records were screened, and 231 records were excluded because they were opinion articles, chapters, case reports, letters to the editor, and studies on burnout in physicians and paramedics, or they used a different version of the scale. Finally, the exact keyword search that considered the human service survey version of the scale was 66. Of these, 31 were consulted and excluded because they did not report means and standard deviations, but other indices were related to burnout, such as correlations, percentage of positive cases, and prevalence. Thus, the present comparative review included 35 studies, of which 19 were conducted on nurses before COVID-19 and 16 during COVID-19, for a total of 77.740 valid cases (see Figure 1-PRISMA flowchart). Throughout the process, the researchers of the current study reviewed the studies based on the inclusion and exclusion criteria, and conflicts were resolved through group discussions.

PICOS-guided eligibility criteria included the following: (P) patient/population: participants were nurses working in critical care units; (I) intervention: studies that applied the MBI to assess burnout reporting means and standard deviations; (C) comparison/study design: burnout in nurses exposed to COVID-19 vs. burnout in nurses before the pandemic; (O) outcome: burnout scores and its three dimensions, such as EE, DP, and PA, categorized as low, moderate, and high (see review Table 2).

**Table 2:** Review results of Maslach Burnout Inventory among nurse EE, DP, and PA means and rating.

**Authors**	**Nation**	**Nurse *N***.	**Unit**	**EE**	**DP**	**PA**	**EE rating**	**DP rating**	**PA rating**
Cao et al. (34)	China	485	Community health nurses	27.40	8.40	25.60	High	Moderate	Low
Cao et al. (35)	China	456	Community health nurses	26.50	8.50	24.60	Moderate	Moderate	Low
Edwards et al. (28)	UK	189	Community mental health	22.32	6.02	31.45	Moderate	Moderate	Low
Faura et al. (18)	Spain	116	Primary care	12.80	6.30	37.90	Low	Moderate	Moderate
Hannigan et al. (24)	UK	283	Community mental health	21.20	5.20	34.80	Moderate	Low	Moderate
Harkin et al. (32)	Ireland	48	Emergency nurses, medical nurse	24.60	11.90	29.10	Moderate	High	Low
Hayter et al. (23)	UK	30	HIV clinical nurses	13.00	15.50	21.30	Low	High	Low
Helps (21)	UK	35	Emergency nurses	36.09	21.34	8.09	High	High	High
Hu et al. (36)	China	420	Community nurses	13.00	15.50	21.30	Low	High	Low
Lorenz et al. (33)	Brasile	168	Primary healthcare	24.60	9.40	30.40	Moderate	Moderate	Low
Poghosyan et al. (30)	USA	13.204	Adult general hospital	22.00	9.40	37.00	Moderate	Moderate	Moderate
Poghosyan et al. (30)	Canada	17.403	Adult general hospital	20.40	8.30	37.20	Moderate	Moderate	Moderate
Poghosyan et al. (30)	UK	9.855	Adult general hospital	19.70	8.90	35.80	Moderate	Moderate	Moderate
Poghosyan et al. (30)	Germany	2.681	Adult general hospital	14.40	7.40	37.10	Low	Moderate	Moderate
Poghosyan et al. (30)	New Zealand	4.799	Adult general hospital	19.80	8.30	37.90	Moderate	Moderate	Moderate
Poghosyan et al. (30)	Japan	5.956	Adult general hospital	25.00	12.40	24.30	Moderate	High	Low
Poghosyan et al. (30)	Russia	442	Adult general hospital	15.10	3.60	20.40	Low	Low	Low
Poghosyan et al. (30)	Armenia	398	Adult general hospital	8.40	3.70	21.90	Low	Low	Low
Quattrin et al. (29)	Italy	100	Oncology	19.50	4.20	38.60	Moderate	Low	Moderate
Schaufeli et al. (20)	Holland	183	General hospital, mental hospital	16.20	5.40	32.70	Low	Low	Low
Schaufeli et al. (20)	Poland	200	General hospital, mental hospital	20.00	8.70	27.30	Moderate	Moderate	Low
Schaufeli et al. (19)	Holland	64	Community nurses	17.50	4.80	30.30	Low	Low	Low
Shmitz et al. (25)	Germany	361	9 Hospital units	10.60	31.00	19.60	Moderate	High	Low
Cámara and Cuesta (27)	Spain	208	Primary care	19.90	7.60	27.40	Moderate	Moderate	Low
Tomàs-Sàbado et al. (31)	Spain	146	Primary care	17.50	4.80	41.20	Low	Low	High
Vahey et al. (26)	Pennsylvania	820	40 Hospital units	24.30	7.40	36.60	Moderate	Moderate	Moderate
Wykes et al. (22)	UK	61	Community nurses	22.50	7.80	35.20	Moderate	Moderate	Moderate
Bellanti et al. (43)	Italy	293	University hospital	26.95	9.09	35.20	Moderate	High	Moderate
Bisesti et al. (46)	Italy	105	SICU	29.10	9.00	32.00	High	Moderate	Low
Chen et al. (44)	China e Taiwan	12.596	Healthcare	19.10	5.50	19.00	Moderate	Moderate	Low
Cortina-Rodriguez and Afanador (38)	Puerto Rico	23	Clinical personnel (Nurses)	32.00	9.80	32.70	High	High	Low
Guixia et al. (37)	China	92	Practical nurses	19.20	5.78	34.45	Moderate	Moderate	Moderate
Hu et al. (45)	China	2.101	ICU	23.40	6.80	34.80	Moderate	Moderate	Moderate
Jakovljevic et al. (50)	Serbia	27	Hospital nurse	30.24	6.85	28.82	High	Moderate	Low
Jalili et al. (41)	Iran	300	Healthcare	26.60	10.20	27.30	Moderate	High	Low
Kakemam et al. (49)	Iran	1.004	Emergency, critical care, general wards	25.94	8.30	29.39	Moderate	Moderate	Low
Kamali et al. (51)	Iran	261	Healthcare	29.22	7.41	18.53	High	Moderate	Low
Murat et al. (42)	Turkey	705	Front-line nurses	11.40	7.30	18.90	Low	Moderate	Low
Pekince et al. (52)	Turkey	270	University hospital	19.30	6.80	19.70	Moderate	Moderate	Low
Rivas et al. (48)	Spain	101	COVID Nurse	32.24	9.51	36.73	High	High	Moderate
Sayilan et al. (40)	Turkey	267	General hospital	23.68	17.14	17.56	Moderate	High	Low
Yörük et al. (47)	Turkey	377	General hospital	20.06	6.42	22.70	Moderate	Moderate	Low
Zhang et al. (39)	China	107	Front-line nurses	12.30	2.10	16.50	Low	Low	Low

The following literature was excluded: conference abstracts, reviews, letters, case reports, posters, unpublished data, and insufficient data, and studies in which averages (e.g., correlation scores or percentages of at-risk cases) were not reported. Health services survey (MBI) data were collected; when data were also collected on other samples, such as physicians and nurses, only data on nurses were reported.

### 2.3. Quality of the studies

The Critical Appraisal tools for use in Joanna Briggs Institute (JBI) Systematic Reviews and the Checklist for Systematic Reviews and Research Syntheses of the JBI Faculty of Health and Medical Sciences at the University of Adelaide, South Australia were used to assess the quality of the studies. The quality of the texts was evaluated by the researchers, and scoring was performed independently. The tool evaluates studies based on 11 standard questions. If the answer was affirmative, the question was assigned a score of 1. If the answer was negative, unclear, or not applicable, a score of 0 was assigned. Studies that scored >8 as an index of study quality and appropriateness were included in this review.

## 3. Results

Table 2 shows the results of the comparative review. The following information was extracted: study characteristics (first author, year of publication, country, number of participants, and type of department) and outcome data [mean emotional exhaustion (EE > 26), mean depersonalization (DP > 9), and mean personal accomplishment (PA < 34)], collected with the MBI. Burnout dimension scores were classified into low, moderate, and high burnout risk according to the MBI scoring guide (see Table 2).

### 3.1. Study characteristics

#### 3.1.1. Before COVID-19

The review included 19 studies that examined 27 samples, comprising 59,111 nurses belonging to departments of Primary Care, Community, General Hospital, Mental Hospital, Emergency, HIV Clinical Care, Community Mental Health, Hospital Units, Oncology, Adult General Hospital, and Medical and Primary Healthcare. Pre–COVID-19 burnout studies were mainly conducted in Europe (eight nations), Asia (three nations), North America (two nations), South America (one nation), and Oceania (one nation). Among the 27 samples, the scores for the EE dimension were classified as low in nine studies, moderate in 16 studies, and high in two studies. While the scores of the DP dimension were classified as low in seven studies, moderate in 14 studies, and high in six studies, the scores of the PA dimension were classified as low in 15 studies, moderate in 10 studies, and high in two studies.

#### 3.1.2. During COVID-19

The studies that detected burnout risk during COVID-19 that met the inclusion criteria were 16, representing 16 samples with 18.629 nurses from COVID-19 departments, emergency, critical care, general wards, front-line, general hospital, university hospital, healthcare, ICU, and SICU. Studies on burnout during COVID-19 were mainly conducted in Asia (four nations), Europe (three nations), and North America (one nation). Among the 27 samples, the scores for the EE dimension were classified as low in two studies, moderate in nine studies, and high in five studies. While the scores of the DP dimension were classified as low in a study, moderate in 10 studies, and high in five studies, the scores of the PA dimension were classified as low in 12 studies and moderate in four studies.

### 3.2. Comparison

#### 3.2.1. Quantitative analysis

**Figure 2:**
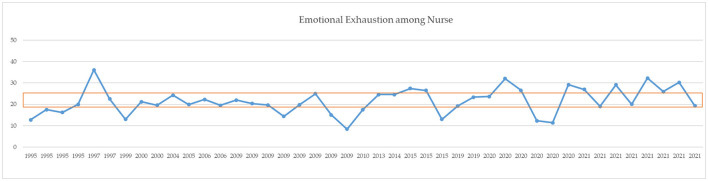
MBI EE means across years. Orange line = moderate range (19–26).

**Figure 3:**
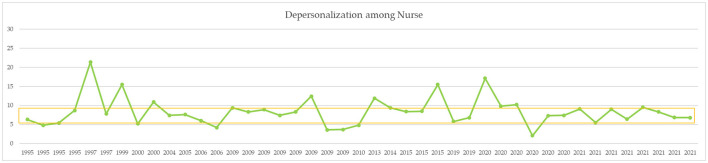
MBI DP means across years. Yellow line = moderate range (6–9).

**Figure 4:**
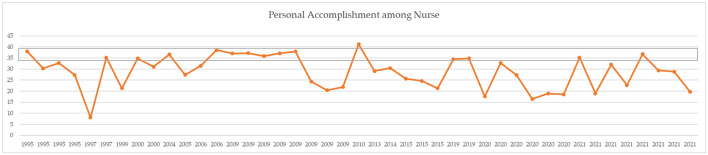
MBI PA means across years. Gray line = moderate range (34–39).

A comparison of means for independent samples was performed with Student's *t*-test comparison of means with 95% confidence intervals. After Bonferroni correction, *p-*values of < 0.01 can be considered statistically significant (Table 3). Mean levels of EE were 20.12 ± 5.63 before COVID-19 and 23.79 ± 6.44 during COVID-19. The DP mean was 8.56 ± 4.03 before COVID-19 and 8.00 ± 3.15 during COVID-19. The PA mean was 30.23 ± 7.58 before COVID-19 and 26.51 ± 7.36 during COVID-19. Comparison of the averages measured did not report statistically significant results (see also the simple boxplot comparing means in Supplementary material). From a qualitative point of view, however, an increase in EE and a decrease in PA are appreciated. Figures 2–4 report the mean of EE, DP, and PA across years. COVID-19 studies started in 2019.

**Table 3:** Statistics about the comparison of the studies before vs. during COVID-19.

**MBI subscales**	**Statistics**	**Scores**
EE	Odds ratio (exp/control)	11.25
	Confidence interval	[1.193, 106.123]
	Left-sided interval	[1.711, +∞]
	Right-sided interval	[−∞, 73.979]
	*P*-value	**0.01**
	Z-score	2.11
DP	Odds ratio (exp/control)	5.83
	Confidence interval	[0.525, 64.823]
	Left-sided interval	[0.193, +∞]
	Right-sided interval	[−∞, 44.014]
	*P*-value	0.07
	Z-score	1.43
PA	Odds ratio (exp/control)	1.60
	Confidence interval	[0.129, 19.838]
	Left-sided interval	[0.193, +∞]
	Right-sided interval	[−∞, 13.235]
	*P*-value	0.357
	Z-score	0.365

The symbol “+∞” represents positive infinity; The symbol “−∞” represents negative infinity.

#### 3.2.2. Qualitative analysis

**Figure 5:**
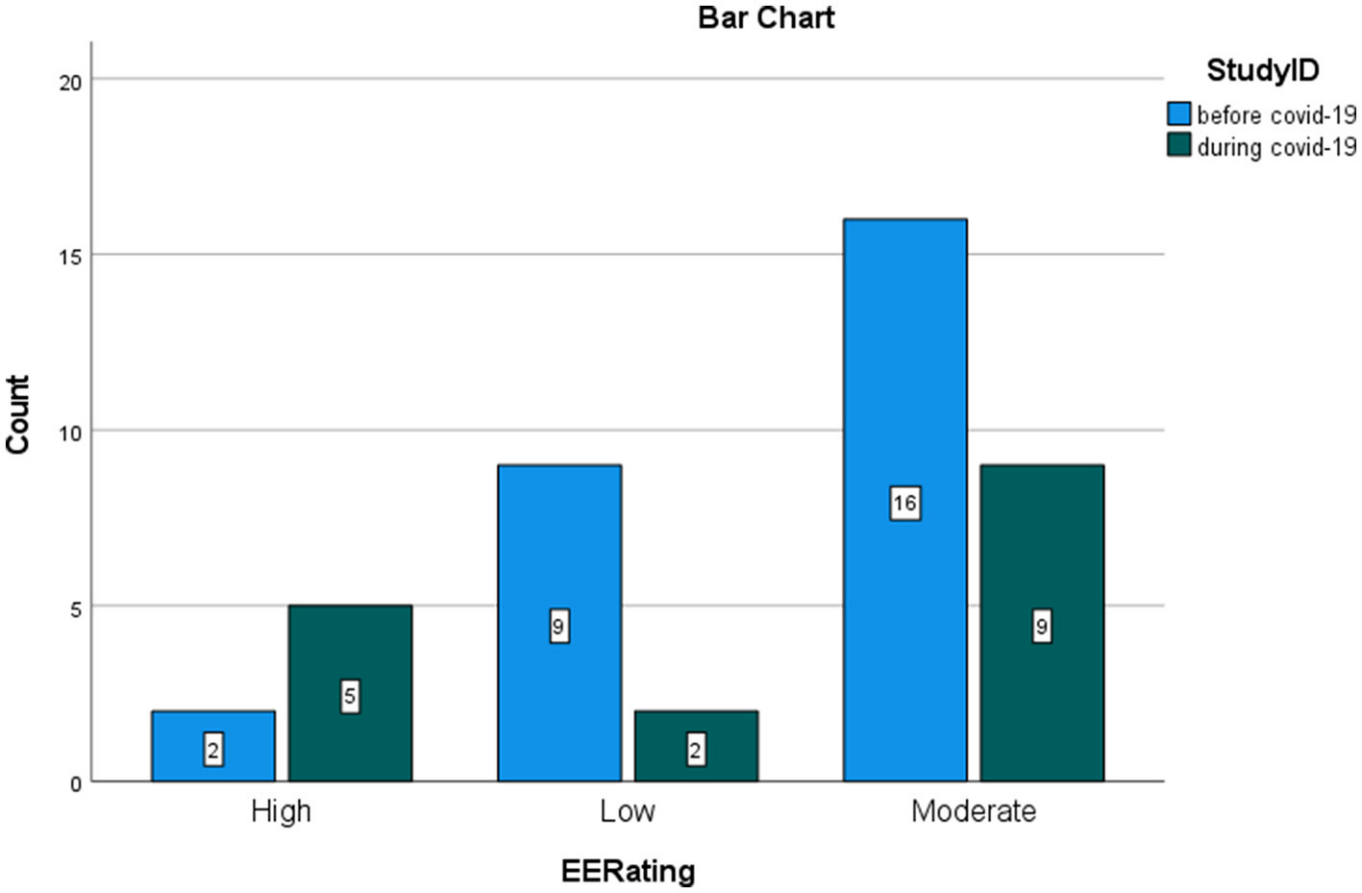
Rating of EE in nurses. Comparison between studies before and during the pandemic.

**Figure 6:**
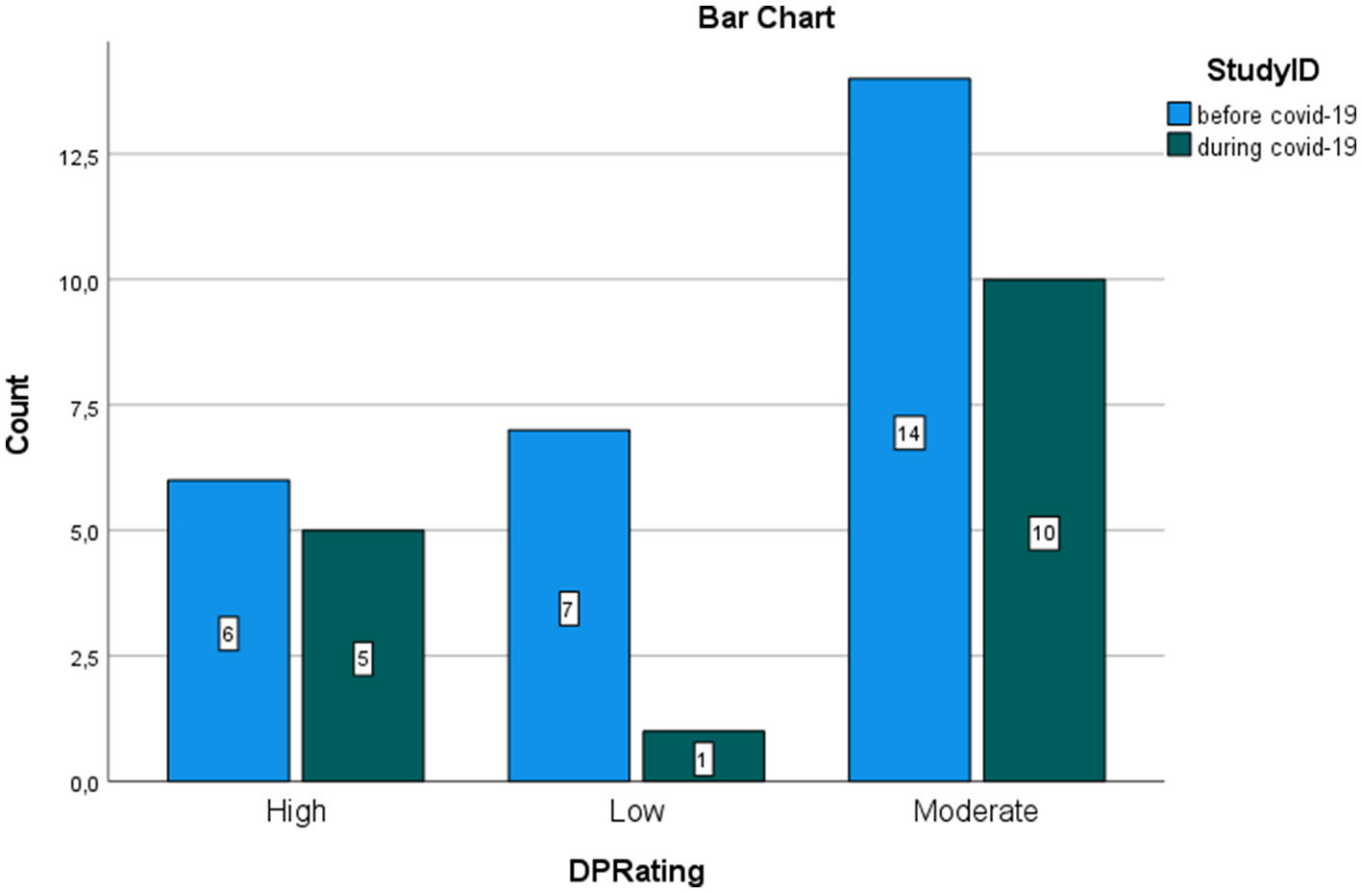
Rating of DP in nurses. Comparison between studies before and during the pandemic.

**Figure 7:**
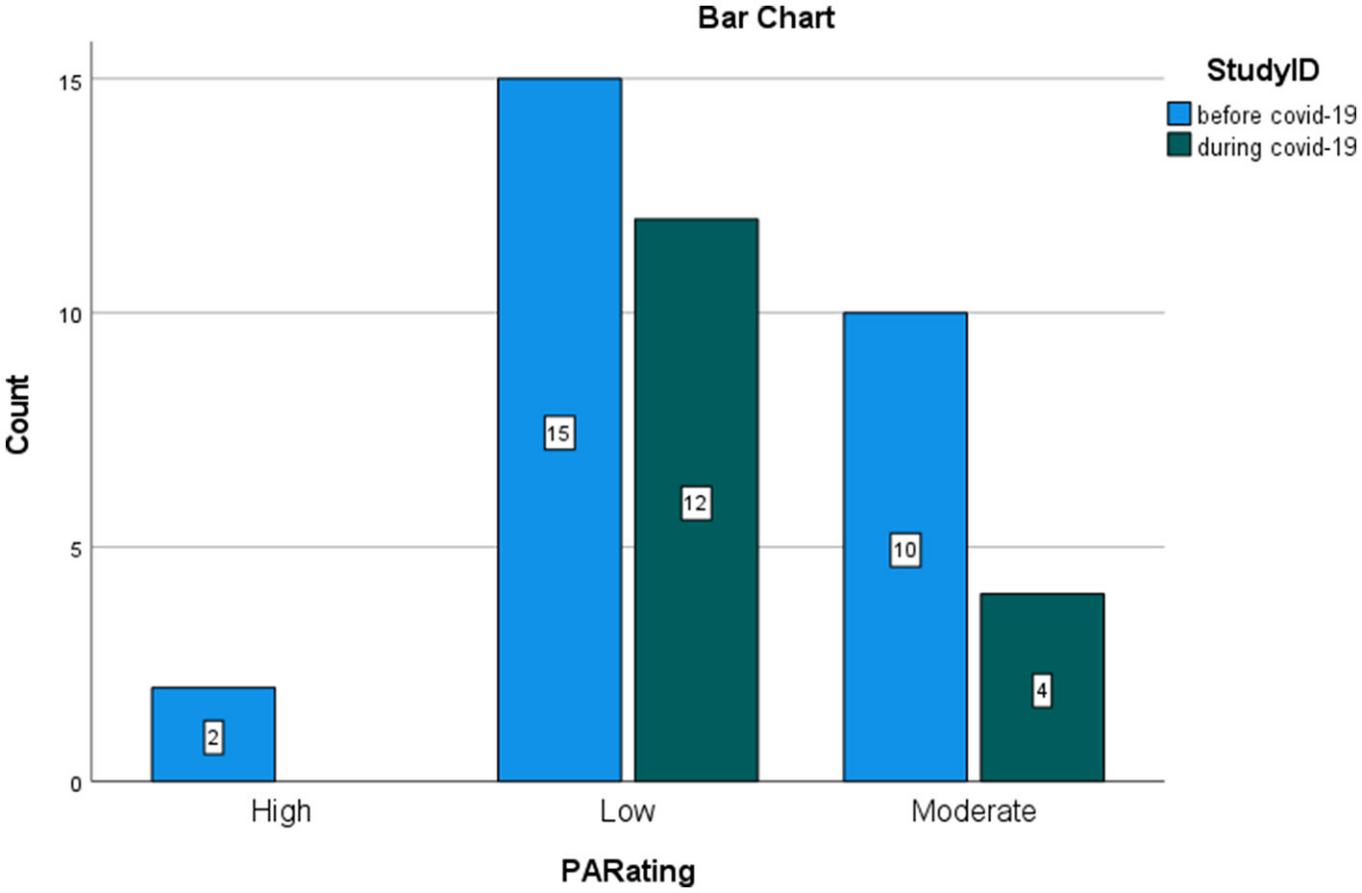
Rating of PA in nurses. Comparison between studies before and during the pandemic.

The graphs represent the comparison of the number of studies that found low, moderate, and high levels of burnout, respectively, before and during COVID-19. MedCalc^®^ odds ratio calculator was used to calculate the ratio between odds, confidence intervals, and *p*-values for the odds ratio (OR) between exposed and control groups. Studies during the COVID-19 pandemic reporting high risk for burnout were considered events in the exposed group. Studies during COVID-19 reporting a low rate of burnout were considered non-events in the exposed group. Studies before the pandemic reporting a high risk of burnout were inserted as events in the control group. Studies before the pandemic reporting low risk were inserted as non-events in the control group. The significance was set at a confidence interval of 95%. Figures 5–7 show the comparison between the number of studies before (*N* = 19) and during (*N* = 16) the COVID-19 pandemic, which found low, moderate, and high EE.

The calculation of the odds ratio shows a significant increase in studies that found high levels of EE during the COVID-19 pandemic, with respect to the studies that were carried out before COVID-19 (Figure 5). On the contrary, the calculation of the odd ratio shows no significant increase in the studies which found high levels of DP during the pandemic, with respect to the studies that were carried out before COVID-19 (Figure 6). Concerning the PA dimension, the calculation of the odd ratio shows no significant increase in the studies which found—in this case—low levels of satisfaction during the pandemic compared with the studies that were carried out before COVID-19 (Figure 7).

## 4. Discussion

### 4.1. Aim of the present study

This comparative review aimed to examine burnout levels among nurses by comparing the average scores of nurses before and during the COVID-19 pandemic. To make the results comparable, the main psychometric assessment tool for burnout was selected for its main factorial components, such as EE, DP, and PA. This comparison was made using a quantitative and qualitative analysis of scores collected from a large number of large samples of health professionals over a period of more than 20 years. To make these data comparable, they were further processed and classified according to risk level: low, moderate, and high (3). Surprisingly, the comparison of the measured average values did not yield statistically significant results. Quantitative findings show that the burnout levels of the nurses were similar before and during the COVID-19 pandemic, whereas qualitative findings show that nurses reported higher levels of EE and lower levels of PA during the COVID-19 pandemic. The distribution of burnout levels across the emotional exhaustion (EE), depersonalization (DP), and personal accomplishment (PA) dimensions differed between the two periods. Before COVID-19, the majority of studies reported moderate scores for EE and DP, with fewer studies reporting high scores. However, during COVID-19, there was an increase in the number of studies reporting high scores for EE and DP. Additionally, during COVID-19, more studies reported low scores for PA compared with the period before COVID-19.

### 4.2. Burnout levels among nurses before the pandemic

High and moderate levels of EE and DP and low levels of PA were already prevalent in the population of nurses in several countries around the world before the pandemic (53, 54). From a theoretical perspective, this finding is consistent with Gee et al. (55) who found that the nursing workforce was already at risk of burnout in previous years. A recent meta-analysis by Ge et al. (56)—including 94 studies covering over 30 countries—revealed that the global prevalence of nursing burnout syndrome over the past 10 years (from 2012 to 2022) was 30.0%, with significant heterogeneity influenced by specialty, region, and year. The prevalence tended to gradually increase during the COVID-19 pandemic, with more significant increases observed in Europe and Africa. Differences in sample size and research scope may account for the discrepancy. Factors contributing to burnout include adverse working conditions such as workload, rotating shifts, low salaries, workplace violence, and a lack of support.

### 4.3. Burnout levels among nurses during the pandemic

During the pandemic, a large percentage of frontline workers had low levels of burnout and a sense of personal satisfaction, also due to role changes from primary and community nurses to frontline workers dealing with patients suffering from COVID-19 (57–59). A study by Dewi et al. (60) found that nurse burnout in Asia during the COVID-19 pandemic was influenced by various factors. Psychological factors such as worry and psychological distress were already significant predictors of burnout (61), while the COVID-19 outbreak has worsened the mental health of healthcare workers, impacting their performance. Religious beliefs and supportive spiritual aspects are important for the mental wellbeing of healthcare workers. Work-related factors such as workload, overtime jobs, and job stress contribute significantly to burnout. Stress in surgical wards and ICUs is particularly associated with burnout among nurses. Insufficient resources and a lack of personal protective equipment (PPE) are additional predictors of stress and burnout. Nurse-patient relationships also play a role in burnout in the form of abuse from patients and emotional situations with the public.

### 4.4. Differences among countries

According to Toscano et al. (62), several studies have highlighted the prevalence of burnout syndrome (BOS) among nurses during the COVID-19 pandemic, taking into account differences among countries. In Belgium, 68% of participating ICU nurses showed BOS symptoms, with emotional exhaustion, depersonalization, and reduced personal accomplishment reported. A Canadian study found moderate-to-high burnout in all nurses, with signs of secondary traumatic stress and intentions to quit. Israeli nurses reported high levels of burnout, which significantly affected professional functioning. Turkish ICU nurses demonstrated a positive correlation between burnout and fear of COVID-19. South African nurses experienced moderate-to-high burnout levels, while Iranian ICU nurses showed emotional exhaustion and depersonalization. Italian nurses exhibited BOS symptoms, with emotional exhaustion being the most prevalent. These findings indicate a relevant risk of BOS among ICU nurses during the pandemic.

In the present study, before COVID-19, studies were more evenly distributed across different continents, with studies conducted in Europe (eight nations), Asia (three nations), North America (two nations), South America (one nation), and Oceania (one nation). During COVID-19, the majority of studies were conducted in Asia (four nations), followed by Europe (three nations) and North America (one nation). The comparison indicates that during the pandemic, there was a notable increase in burnout levels among nurses in Asian countries, with more studies reporting high scores in emotional exhaustion and depersonalization. Additionally, there was a general increase in studies reporting low scores in personal accomplishment during COVID-19, indicating potential challenges in maintaining a sense of achievement and fulfillment among nurses during the pandemic.

Europe continued its research efforts during the pandemic, but burnout levels remained relatively consistent before COVID-19. North America had a reduced focus on burnout research during the pandemic. However the available data from this region showed similar patterns of increased high scores in EE and DP and more studies reporting low scores in PA during COVID-19.

### 4.5. Limitations

The study also has some limitations. In interpreting the results, it is necessary to take into account the differences (e.g., healthcare systems, working hours, and work–life balance) in terms of the countries in which the studies were conducted and the year of their publication. Regarding possible publication bias, it is necessary to consider the possible bias of the studies included in the study, a factor that was attempted to be controlled by critical reading of the studies before their inclusion. A standard meta-analytic methodology was not used due to a lack of statistical parameters, yet it could be well performed if the amount of research data allowed it. Because the articles reviewed were descriptive studies, the level of evidence from the studies is low, but it is sufficient to analyze means, standard deviations, and percentages and to relate the variables and their integration in a comparative review such as the one conducted here. Furthermore, the comparison of burnout between the two groups might be affected by differences in sample size. The group with a larger sample size (before COVID-19) will likely have higher statistical power to detect smaller effects, while the group with a smaller sample size (during COVID-19) may have reduced statistical power to detect significant differences. Finally, it would have been ideal to compare the same subjects under different conditions, but unfortunately, there are no longitudinal studies on burnout levels.

## 5. Conclusion

The present study has shown that burnout is already a silent epidemic that is certainly exacerbated by COVID-19. However, considering the literature data, COVID-19 can only explain a portion of the burnout levels among nurses. The literature accurately shows how these healthcare professionals had already been in a precarious situation for years (61). Addressing burnout among nurses should remain a top priority. Implementing comprehensive support systems, enhancing work–life balance, and fostering a positive work environment can help mitigate burnout risk (62). Longitudinal studies should investigate the lasting effects of the pandemic on nurse burnout. Tailored interventions, training programs, and mental health resources can aid nurses in coping with the challenges they face (63–65). Continuous monitoring and preventive strategies are essential to safeguarding the wellbeing of healthcare professionals (66–71).

## Data availability statement

The original contributions presented in the study are included in the article/supplementary material, further inquiries can be directed to the corresponding authors.

## Author contributions

AR and FC: conceptualization, software, and investigation. AR: methodology and data curation. AC and MY: validation. FC: formal analysis. AC: resources, visualization, supervision, and project administration. AR, MY, and GGÖ: writing—original draft preparation. MY, FC, GGÖ, ŁS, SZ, and GN: writing—reviewing and editing. All authors contributed to the article and approved the submitted version.
